# Contribution of PGE_2_ EP1 receptor in hemin-induced neurotoxicity

**DOI:** 10.3389/fnmol.2013.00031

**Published:** 2013-10-07

**Authors:** Shekher Mohan, Alexander V. Glushakov, Alexander deCurnou, Shuh Narumiya, Sylvain Doré

**Affiliations:** ^1^Department of Anesthesiology, Center for Translational Research in Neurodegenerative Disease, University of FloridaGainesville, FL, USA; ^2^Department of Pharmacology, Kyoto UniversityKyoto, Japan; ^3^Departments of Neuroscience, Neurology and Psychiatry, University of FloridaGainesville, FL, USA

**Keywords:** calcium, GPCR, heme, neuroinflammation, prostaglandin

## Abstract

Although hemin-mediated neurotoxicity has been linked to the production of free radicals and glutamate excitotoxicity, the role of the prostaglandin E_2_ (PGE_2_)-EP1 receptor remains unclear. Activation of the EP1 receptor in neurons results in increased intracellular calcium levels; therefore, we hypothesize that the blockade of the EP1 receptor reduces hemin neurotoxicity. Using postnatal primary cortical neurons cultured from wild-type (WT) and EP1^−/−^ mice, we investigated the EP1 receptor role in hemin neurotoxicity measured by lactate dehydrogenase (LDH) cell survival assay. Hemin (75 μM) induced greater release of LDH in WT (34.7 ± 4.5%) than in EP1^−/−^ (27.6 ± 3.3%) neurons. In the presence of the EP1 receptor antagonist SC-51089, the hemin-induced release of LDH decreased. To further investigate potential mechanisms of action, we measured changes in the intracellular calcium level [Ca^2+^]_i_ following treatment with 17-phenyl trinor PGE_2_ (17-pt-PGE_2_) a selective EP1 agonist. In the WT neurons, 17-pt-PGE_2_ dose-dependently increased [Ca^2+^]_i_. However, in EP1^−/−^ neurons, [Ca^2+^]_i_ was significantly attenuated. We also revealed that hemin dose-dependently increased [Ca^2+^]_i_ in WT neurons, with a significant decrease in EP1^−/−^ neurons. Both 17-pt-PGE_2_ and hemin-induced [Ca^2+^]_i_ were abolished by N-methyl-D-aspartic (NMDA) acid receptor and ryanodine receptor blockers. These results suggest that blockade of the EP1 receptor may be protective against hemin neurotoxicity *in vitro*. We speculate that the mechanism of hemin neuronal death involves [Ca^2+^]_i_ mediated by NMDA acid receptor-mediated extracellular Ca^2+^ influx and EP1 receptor-mediated intracellular release from ryanodine receptor-operated Ca^2+^ stores. Therefore, blockade of the EP1 receptor could be used to minimize neuronal damage following exposure to supraphysiological levels of hemin.

## Introduction

Heme is a protein prosthetic group consisting of a porphyrin ring and an iron atom in the center (Padmanaban et al., 1989). Heme is most commonly recognized as vital component of hemoglobin (Hb). The mitochondria are the main site for heme synthesis, and heme is essential for mitochondrial function. After its synthesis in the mitochondria, heme can migrate across lipid-water interfaces and hence diffuse rapidly from the mitochondrial inner membrane (site of synthesis) to the rough endoplasmic reticulum where it is incorporated into Hb. Under physiological conditions, free heme is rarely detected. In hemoproteins, heme is bound to aromatic amino acid, such as phenylalanine, tyrosine, or tryptophan, allowing for its hydrophobic properties (Li et al., 2011). Also, under normal physiological conditions, the bond between heme and Hb is very stable; hence, the amount of free heme is expected to be negligible. However, in aged or pathological cells, Hb becomes oxidized and denatured, weakening the bond between heme and Hb; thus the cellular effects of free heme are increased (Shaklai et al., 1985). Under pathophysiological conditions high concentrations of free heme can also enter cells by heme-transporters (Krishnamurthy and Schuetz, 2005; Azuma et al., 2008).

The biological function of heme stems from the iron atom bound in the central porphyrin ring of the molecule. Iron is redox-active and can readily accept or donate electrons, i.e., ferrous iron (Fe^2+^) is oxidized to ferric iron (Fe^3+^). However, under pathophysiological conditions, disruptions in iron homeostasis can cause an increase in the oxidation of iron from Fe^2+^ to Fe^3+^ via the Fenton reaction. The increase in Fe^3+^ concentration can produce harmful superoxide free radicals in the brain that lead to oxidative stress and neuronal death (Smith and Perry, 1995; Valko et al., 2005). Hemolysis of erythrocytes releases Hb into the extracellular space and becomes oxidized from ferrous to ferric Hb, which in turn causes the release of heme (Umbreit, 2007). When internalized by cells, free heme is catabolized by heme oxygenases (HO1 and HO2 isoforms) into hemin (Balla et al., 1993; Ferris et al., 1999). HO2 is constitutively expressed and catabolizes heme at homeostatic conditions (Trakshel et al., 1986). However, excess heme induces the expression of the stress-responsive HO1 isoform, which results in increased heme breakdown, and under physiological conditions, prevents its putative neurotoxic effects (Ferris et al., 1999; Wang and Doré, 2007; Gozzelino et al., 2010). Similarly, when activated under physiological conditions, the HO2 isoform converts hemin to iron, biliverdin and bilirubin which have been found to be neuroprotective against oxidative stress (Doré et al., 1999). Supraphysiological levels of hemin are neurotoxic due to its ability to contribute to the production of reactive oxygen species (ROS) which are mediated by the redox iron (Kumar and Bandyopadhyay, 2005). Hemin can also cause endothelial injury and has pro-inflammatory properties that create pathology in multiple diseases. For example, hemin released from lyzed erythrocytes can contribute to brain damage as seen after hemorrhagic stroke (Regan et al., 2001, 2002, 2004; Goldstein et al., 2003).

Recent evidences suggest that the accumulation of intact hemin itself is more neurotoxic than the iron released from its breakdown (Dang et al., 2011). Here, we present data that may provide further evidence for the basis of hemin-mediated neurotoxicity. In addition, a recent study provided evidence for iron accumulation mediated by inflammatory stimuli in neurons and microglia, suggesting that neuroinflammation results in intracellular iron accumulation and oxidative damage (Urrutia et al., 2013). Therefore, further studies on the cross-talk between neuroinflammatory mediators such as prostaglandins and iron-containing hemoproteins or hemin are warranted.

Prostaglandins, via their specific G protein coupled receptors, have a variety of physiological functions in the central nervous system. The major prostaglandin, prostaglandin E_2_ (PGE_2_) is produced by various cell types and is involved in ischemic neuronal injury (Doré et al., 2003; Li et al., 2008). We and others have found that targeting different types of prostaglandin receptors by selective blockade or activation protects against neurotoxicity and ischemic stroke (Echeverria et al., 2005; Ahmad et al., 2006a,b; Doré, 2006). The up-regulation of cyclooxygenase-2 (COX-2) and greater PGE_2_ levels together contribute to N-methyl-D-aspartic acid (NMDA)-induced neuronal injury (Hewett et al., 2000; Ahmad et al., 2006a, 2008). Activation of these PGE_2_ receptors can regulate intracellular levels of cyclic adenosine monophosphate and/or calcium ([Ca^2+^]_i_) (Kim et al., 2002; Linhart et al., 2003). The effects of PGE_2_ are regulated by its respective affinity for its receptors and their relative abundance, which we recently reviewed (Mohan et al., 2012). Although the neurotoxicity of hemin has been previously demonstrated, the role of prostaglandins and their cognate receptors in hemin-induced neurotoxicity remains not fully understood. Using *in vivo* and *in vitro* experiments, activation of the EP1 receptor has been reported to be consistently involved in PGE_2_-mediated neurotoxicity (Lee et al., 2004; Carrasco et al., 2007; Ahmad et al., 2008). We present here functional data that supports previous reports on the role of the EP1 receptor in neurotoxicity. Using genetic and pharmacological tools, we hypothesize that EP1 receptor-mediated signaling potentiates hemin-mediated cytotoxicity in cortical neurons. We first investigated expression of EP1 and other PGE_2_ receptor subtypes in primary cultured cortical neurons. Next, we determined what concentration of hemin would produce neurotoxicity and we addressed the importance of the EP1 receptor. Then, to elucidate functional changes we measured changes in Ca^2+^ signaling in response to the EP1 receptor agonist, 17-pt-PGE_2_ and hemin with and without the pharmacological blockers used to determine the source of calcium. This is the first known study to measure the effect of EP1 receptor in hemin-mediated neurotoxicity and [Ca^2+^]_i_ in primary cortical neuronal culture.

## Materials and methods

All animal protocols were approved by the Institutional Animal Care and Use Committee of the University of Florida. All mice were maintained and housed in the University's core facilities under controlled conditions with *ad libitum* access to food and water.

### Preparation of postnatal primary cortical neuronal cultures

Postnatal mouse neuronal cultures were isolated from 0- to 1-day-old WT and EP1^−/−^ pups, cultured in serum-free Neurobasal medium supplemented with GlutaMax (Life Technologies, Grand Island, NY), and NeuroCult SM1 (STEMCELL Technologies, Vancover, BC) and plated onto poly-D-lysine-coated 24-well plates at a density of 5 × 10^5^ cells/well. Cells were maintained in growth medium at 37°C in 95% air/5% CO_2_-humidified incubator for 10–12 days *in vitro* before treatment. Fifty percent of the media was exchanged with fresh medium containing B27 (Life Technologies, Grand Island, NY) every 4 days. Neurons from WT and EP1^−/−^ pups were treated with either vehicle control or hemin (Frontier Scientific, Logan, UT) in Neurobasal/B27 minus antioxidant supplemented medium. For direct comparison between WT and EP1^−/−^ neuronal cultures, “sister cultures” were used to increase the reliability of our data.

### Absolute quantitative real-time PCR (qRT-PCR)

DNA vectors (pANT7_cGST, from DNASU) containing inserts for each receptor (EP1-4) were cultured overnight in Luria-Bertani broth/Amp (100 μg/mL) at 37°C. Plasmid DNA was purified with a QIAprep Spin Miniprep Kit (Qiagen, Valencia, CA). Purified bacterial plasmid DNA was linearized through restriction enzyme digestion using *Nco1* (New England Biolabs, Ipswich, MA, USA) using manufacturer protocols. Completion of plasmid digestion was visualized through gel electrophoresis with ethidium bromide staining. Linearized plasmid DNA was gel extracted using the Qiagen gel extraction kit (Qiagen) following the manufactures protocols and quantified via Nanodrop-1000 spectrophotometer (Thermo Scientific, Waltham, MA). To analyze the copy number of each receptor, eight point standard curves ranging from 4.0 × 10^7^ to 4000 copies per μL was constructed using linearized plasmid DNA obtained from each receptor. Finally, to ascertain the best concentration of cDNA synthesized from the RNA isolated from our neuronal cultures and determine copy numbers, a six point standard curve was developed for all four receptors ranging from 200 to 3.125 ng of total cDNA. The copy numbers for each receptor type was estimated using the following formula: number of copies/μL = 6.022 × 1023 (moles/mole) × DNA concentration (g/μL)/number of base pairs × 660 Dalton's (Godornes et al., 2007). From our neuronal cultures, total RNA was isolated using the PureLink RNA Mini Kit as detailed in the manufacture's manual (Life Technologies). One microgram of total RNA was subjected to DNase I treatment and concentration determined using the Nanodrop-1000 spectrophotometer and A260/A280 ratio (1.8–2.1) recorded (Thermo Scientific). One microgram of total RNA was reverse transcribed using the High-Capacity cDNA reverse transcription cDNA kit as described by the manufacturer's protocol (Life Technologies). The following mouse specific TaqMan Gene Expression Assays for EP1-4 was used: (assay ID no's, Mm00443098_g1; Mm00436051_m1; Mm01316856_m1; Mm00436053_m1) (Life Technologies). The qRT-PCR amplification cycle was: 95°C for 3 min followed by 39 cycles of 95°C for 10 s 60°C for 30 s using the BioRad C1000 thermal cycler.

### Hemin neurotoxicity experiments and assessment of cell survival

To assess hemin neurotoxicity, neurons were treated with control (Neurobasal/B27 minus antioxidant supplement medium containing 0.05% 0.1M NaOH) and hemin at concentrations 0, 12.5, 25, 50, 75, and 100 μM for 18 h. In separate sets of experiments, neurons were pre-treated with EP1 receptor selective antagonist SC-51089 (10 μM) (Cayman Chemical, Ann Arbor, MI) and selective COX-2 inhibitor NS-398 (10 μM) (Sigma-Aldrich) and NMDAR antagonist MK801 (10 μM) (Sigma-Aldrich, St. Louis, MO), for 15 min in Neurobasal/B27 minus antioxidant supplement media and then with these agents still present, neurons were co-treated with vehicle or hemin (75 μM) for 18 h. Cell viability was assessed using the CytoScan™-Fluoro assay (G-Biosciences, St. Louis, MO). This is a fluorometric assay (excitation/emission 530/590 nm) for estimating cell cytotoxicity based on the release of LDH from cells with damaged membranes. The low LDH activity (background) in wells subjected to medium exchange only was subtracted from all values to yield the signal specific for the neurotoxic insult-mediated by hemin as described by Koh and Choi (Koh and Choi, 1987). To assess the number of live cells, the Calcein AM (Life Technologies) assay was performed as instructed by the manufactured protocol. Briefly, following treatment, medium was removed, neurons were gently rinsed once in 1× DPBS and 200 μL of Calcein AM (3 μM) added to neurons for 1 h at 37°C. Next, the wells were gently rinsed twice with 1× DPBS and the fluorescence from each well was measured using the FlexStation 3 microplate reader (Molecular Devices) at excitation/emission 485/530 nm. Micrographs of neurons were acquired using a 20× DIC objective on a Leica DMI6000 B monochrome digital camera and captured using the MetaMorph software (Molecular Devices LLC, Sunnyvale, CA).

### Calcium imaging

Eight to ten-day-old postnatal mouse cortical neuronal cultures were loaded with 3 μM Quest Fluo-8™ (Fluo-8 AM) (ATT Bioquest, Inc, Sunnyvale, CA) in Hanks' balanced salt solution (HBSS) without CaCl_2_ and MgCl_2_ (Life Technologies) for 1 h and washed twice with HBSS consisting of 2 mM CaCl_2_ and 1 mM MgCl_2_. The membrane-permeable fluorescent calcium indicator Fluo-8 AM was used for imaging dynamic changes in [Ca^2+^]_i_, an increase in fluorescence intensity of Fluo-8 AM corresponds to an increase in [Ca^2+^]_i_. To determine if the change in fluorescence following treatment with hemin reached plateau, calibration of Fluo-8 AM was performed with NMDA (100 μM) and ionomycin (5 μM) after the addition of hemin in the same cultures. Changes in Fluo-8 AM-fluorescence intensity were measured following addition of 17-pt-PGE_2_ (100 nM and 1 μM) (Cayman Chemical, Ann Arbor, MI) or hemin (1–25 μM) for 60 s with and without 15 min pre-treatment with SC-51089 (10 μM), NS-398 (10 μM), MK-801 (10 μM), nifedepine (10 μM), ryanodine (50 μM) and vehicle (0.05% DMSO). The intensity of fluorescence was determined with excitation 482 nm and emission 505–530 nm of selected neurons using a Leica DMI6000 B inverted microscope equipped with Leica HC PL FLUOTAR 20×/0.50 objective and Lambda DG-5 Plus. All images were captured using a Hamamatsu electron multiplying charged-coupled device camera and select regions acquired at a scanning time of ~1 s per frame. A time course of changes in fluorescence intensity generated was with MetaFluor Fluorescence imaging software (Molecular Devices, LLC). Fluo-8 AM fluorescence values are presented as Δ *F/F*_0_ where Δ *F* = *F* − *F*_0_, *F* is the fluorescence recorded following 17-pt-PGE_2_ or hemin application and *F*_0_ is the mean basal fluorescence. To determine statistical analysis between treatments, area under the curve (AUC) was calculated for each neuron recorded using the trapezoid rule as computed by GraphPad Prism. Data for each treatment was from 3 to 6 independent neuronal cultures. All imaging experiments were done at room temperature.

### Statistics

One-Way ANOVA was used to test for differences in mean values from multiple samples (LDH and Calcein AM assays and calcium imaging data) with Bonferroni's multiple comparison tests. Statistical difference between two means was analyzed by two-tailed unpaired or paired Student's *t*-test. Differences were considered significant if *p* < 0.05. All data are expressed as mean ± SEM. All data were analyzed by GraphPad Prism 6.0 software.

## Results

### Neuronal cultures express PGE_2_ EP1–4 receptors

**Figure 1:**
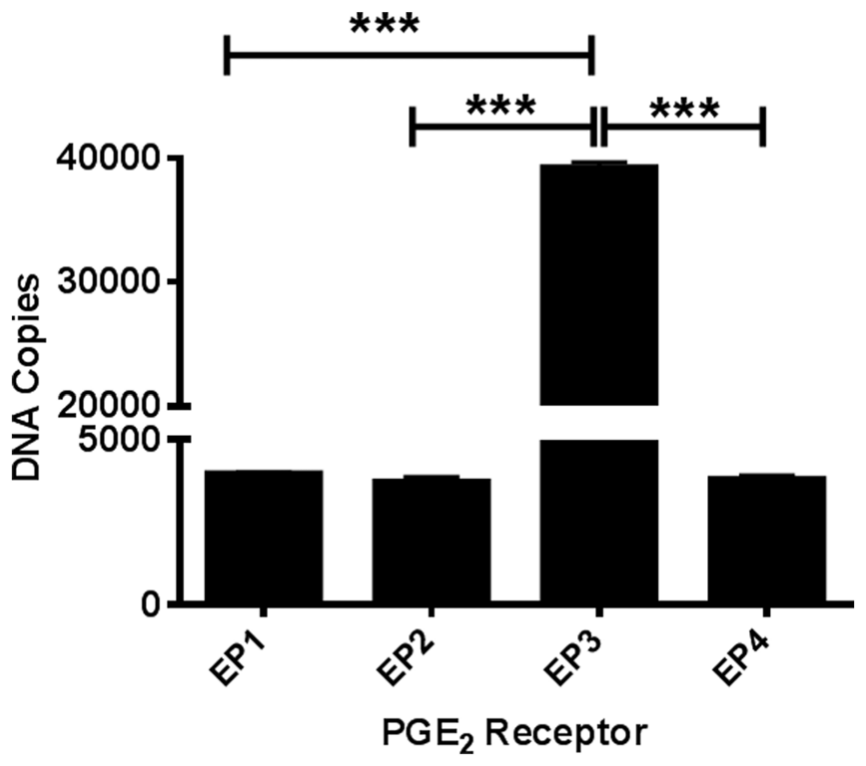
**PGE_2_ EP1-4 receptors are expressed in cultured cortical neurons.** Absolute qRT-PCR was performed using mouse-specific TaqMan assays to measure basal expression of EP1-4 receptors in postnatal neuronal cultures. The expression of the receptors EP1 through EP4 mRNA is presented as estimated DNA copies using linearized plasmid standards for each PGE_2_ receptor. Data represents mean ± SEM of triplicate measures from four neuronal cultures. Statistical analysis was carried out using One-Way ANOVA, with Bonferroni's multiple comparison tests. ^***^p < 0.001.

Due to the lack of selectivity of antibodies against the EP1-4 receptors, we measured basal mRNA levels in our postnatal cortical neuronal cultures. All four EP receptors are expressed at the mRNA level in our neuronal cultures. Using absolute qRT-PCR with gene-specific TaqMan assays we calculated the copy numbers of each PGE_2_ receptor subtype. EP3 (estimated 40,000 copies) is the most highly expressed receptor. The mRNA expression of EP1, EP2, and EP4 receptors all had the same levels of expression (estimated 4000 copies) (Figure 1).

### Knockout and blockade of EP1 receptor is protective against hemin neurotoxicity

**Figure 2:**
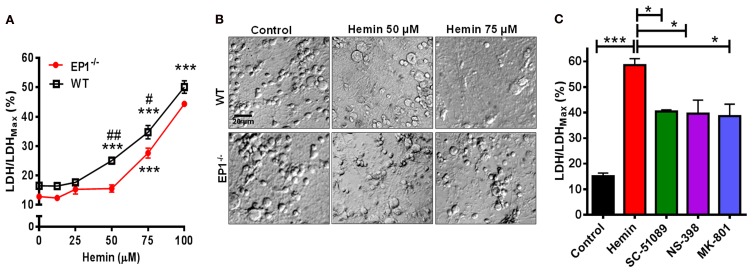
**EP1 receptor blockade and knockout reduces hemin neurotoxicity.** Primary postnatal neurons from WT and EP1^−/−^ pups cultured in serum-free Neurobasal media were treated with hemin (12.5–100 μM) for 18 h. **(A)** Hemin-induced cell viability was measured by LDH assay [LDH/LDH_Max_ %] from WT and EP1^−/−^ neurons. ^***^p < 0.001 vs. control; ^#^*p* < 0.01 and ^##^*p* < 0.001 compared to the effect of hemin (50 and 75 μM) in neurons cultured from EP1^−/−^ pups. **(B)** Cultured WT and EP1^−/−^ neurons were treated with vehicle control or hemin (50 and 75 μM) for 18 h and phase-contrast images were captured using a 20× DIC objective. **(C)** Cultured WT neurons were pre-treated with EP1 receptor antagonist, SC51089 (10 μM), COX-2 antagonist, NS-398 (10 μM) and NMDAR antagonist, MK-801 (10 μM) for 15 min and were then co-treated with hemin (75 μM). After 18 h of treatment, cell viability was measured using LDH assay. LDH assay data represent means ± SEM of duplicate measures from triplicate wells from four to five experiments. Statistical analysis was carried out using One-Way ANOVA, with Bonferroni's multiple comparison tests. ^***^p < 0.001 vs. control; ^*^p < 0.05 vs. hemin alone.

Using neuronal cultures derived from WT and EP1^−/−^ mouse pups, hemin dose-response experiments were performed. In neurons from WT pups, hemin caused significant neurotoxicity as measured using the CytoScan-Fluoro LDH assay. LDH levels released by 50 μM (25.0 ± 2.2%), 75 μM (34.7 ± 4.5%), and 100 μM (50.1 ± 4.2%) of hemin were significantly greater compared to control (16.5 ± 1.5%). Using the LDH assay, we also found hemin to be neurotoxic in neurons cultured from EP1^−/−^ mice. Hemin at 75 μM (27.6 ± 3.3%) and 100 μM (44.3 ± 0.8%) significantly increased LDH release as compared to control (12.8 ± 1.7) (Figure 2A). However, the percentage of LDH released in response to hemin (50 and 75 μM) was significantly less in EP1^−/−^ neurons compared to WT neurons. In separate experiments, WT neurons were pre-treated with NMDAR antagonist, MK-801 (10 μM) or EP1 receptor antagonist SC-51089 (10 μM) for 15 min and then co-treated with hemin (75 μM). Compared to hemin alone (61.8 ± 1.9%), MK-801 and SC-51089 significantly decreased (38.6 ± 10.4% and 42.7 ± 2.8%, respectively) hemin-induced LDH release (Figure 2C). Similarly, treatment with the COX-2 selective antagonist NS-398 significantly decreased hemin-induced LDH release. This data suggests that EP1 receptor, and its upstream COX-2 signaling pathways as well as NMDAR are involved in hemin neurotoxicity.

**Figure 3:**
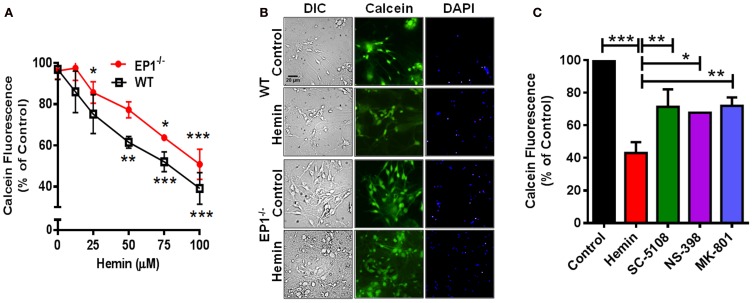
**EP1 receptor blockade increased neuronal survival following hemin treatment.** Primary postnatal neurons from WT and EP1^−/−^ pups cultured in serum-free Neurobasal media were treated with hemin (12.5–100 μM) for 18 h. **(A)** Neuronal survival following hemin treatment was measured by Calcein AM assay (% of control) from WT and EP1^−/−^ neurons. ^***^p < 0.001, ^**^p < 0.01 and ^*^p < 0.05 vs. control. **(B)** Cultured WT and EP1^−/−^ neurons were treated with vehicle control or hemin (75 μM) for 18 h and phase contrast and fluorescence images were captured with a 20X phase-contrast images using a 20× DIC objective. **(C)** Cultured WT neurons were pre-treated with SC51089 (10 μM), NS-398 (10 μM) and MK-801 (10 μM) for 15 min and then co-treated with hemin (75 μM). After 18 h of treatment, live cells were measured using the Calcein AM assay. Calcein AM assay data represent means ± SEM of duplicate measures from triplicate wells from four experiments. Statistical analysis was carried out using One-Way ANOVA, with Bonferroni's multiple comparison tests. ^***^p < 0.001 vs. control; ^**^p < 0.01 and ^*^p < 0.05 vs. hemin alone.

In addition to measuring the LDH content, Calcein AM assay was performed to measure the number of live cells. In separate cohort of neuronal cultures, hemin dose-dependently also decreased the number of live neurons; however the number of live neurons was greater in EP1^−/−^ neurons as compared to WT neuronal cultures when treated with 50 and 75 μM of hemin (Figure 3A). In addition to decreasing hemin-induced LDH release, treatment with SC-51089 also reduced the number of dead cells after hemin treatment. Compared to hemin alone (43.1 ± 6.6%), the number of Calcein AM positive live neurons increased (71.4 ± 10.7%) following treatment with SC-51089. Similarly, following treatment with NS-398 and MK-801, the number of live neurons significantly increased compared to hemin treatment alone (Figure 3C). In addition, hemin induced a change in the appearance of neurons from WT and EP1^−/−^ mice as compared to control (Figures 2B, 3B). Representative images show changes in hemin-induced Calcein AM fluorescence (live cells) and diamidino-2-phenylindole (DAPI) staining in WT and EP1^−/−^ neuronal cultures (Figure 3B).

### EP1 receptor-mediated [Ca^2+^]_i_

**Figure 4:**
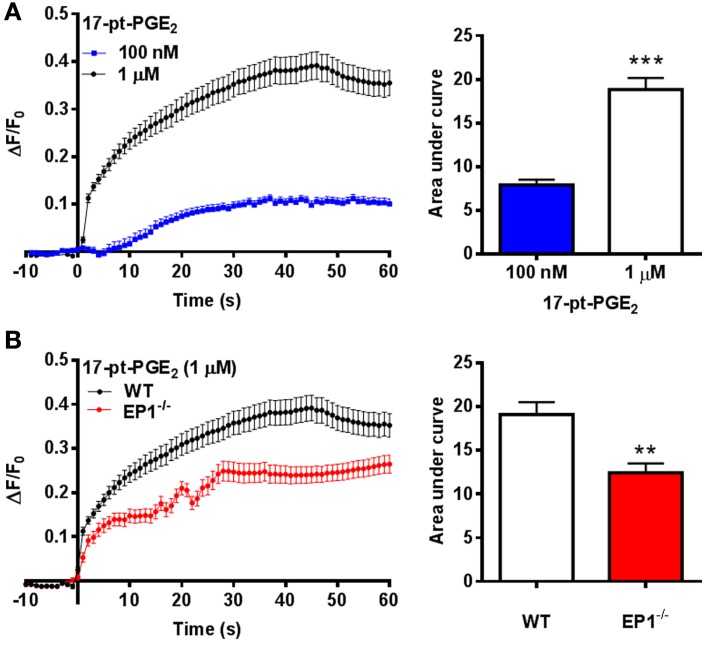
**EP1 receptor mediated changes in [Ca^2+^]_i_ in neurons. (A)** Δ *F/F*_0_ values recorded in response to 17-pt-PGE_2_ (100 nM and 1 μM) normalized to the fluorescence values recorded before application of 17-pt-PGE_2_ and plotted as a function of time. Bar charts represent changes in AUC (arbitrary units) following treatment with 17-pt-PGE_2_. **(B)** Δ *F/F*_0_ values recorded in response to 17-pt-PGE_2_ (1 μM) in neurons cultured from WT and EP1^−/−^ pups. Data represent mean ± SEM. Statistical analysis was carried out using unpaired students *t*-test ^***^p < 0.001 to compare the change in fluorescence intensity between different two groups: 100 nM vs. 1 μM; ^**^p < 0.01 to compare between genotypes WT vs. EP1^−/−^ in response to 17-pt-PGE_2_.

The mechanism responsible for hemin neurotoxicity is not well understood. Glutamate excitotoxicity, a process that involves Ca^2+^ overload is suggested to be responsible for neuronal death following hemin treatment. Thus, as an alternative mechanism, we investigated EP1 receptor-associated [Ca^2+^]_i_ in cortical neurons. Treatment with 17-pt-PGE_2_ (100 nM and 1 μM) significantly increased [Ca^2+^]_i_ (AUC: 100 nM—7.9; 1 μM—18.8) (Figure 4A). Next, 17-pt-PGE_2_-induced [Ca^2+^]_i_ was compared between WT and EP1^−/−^ neuronal cultures. The mean AUC following application of 17-pt-PGE_2_ (1 μM) was significantly different between WT and EP1^−/−^ neurons (18.8 vs. 12.4 respectively). (*p* < 0.01, unpaired Student's *t*-test) (Figure 4B).

**Figure 5:**
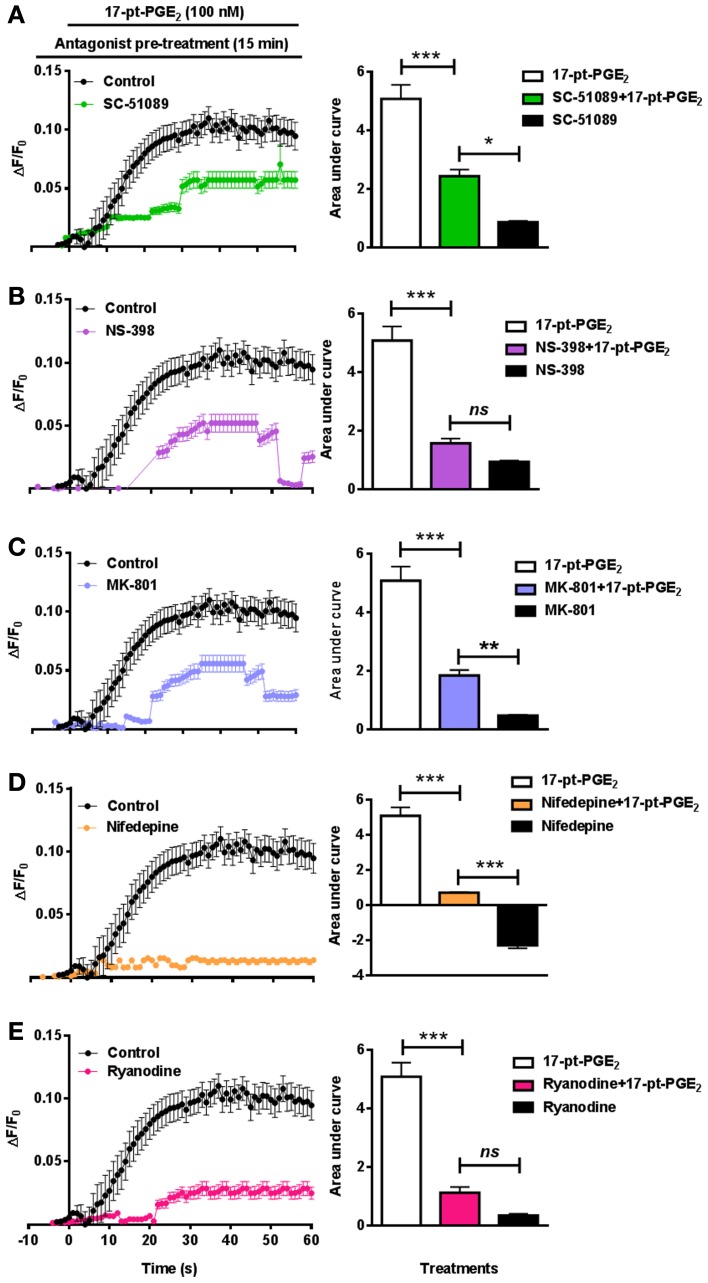
**Characterization of EP1 receptor mediated changes in [Ca^2+^]_i_ in neurons.** In a separate set of neuronal cultures, neurons were pre-treated with SC-51089 (10 μM), NS-398 (10 μM), MK-801 (10 μM), nifedepine (10 μM), ryanodine (50 μM) or vehicle control before 17-pt-PGE_2_ (100 nM) application and fluorescence intensity was recorded. Bars above graphs indicate application time of 17-pt-PGE_2_ and antagonists/vehicle control. **(A–E)** Δ *F/F*_0_ and changes in AUC induced by 17-pt-PGE_2_ alone and following pre-treatment with **(A)** SC-51089, **(B)** NS-398, **(C)** MK-801, **(D)** nifedepine, and **(E)** ryanodine. Data represent mean ± SEM. Statistical analysis was carried out using One-Way ANOVA with Bonferroni's multiple comparison tests. ^***^p < 0.001 for17-pt-PGE_2_ alone or 17-pt-PGE_2_ plus antagonist. ^**^p < 0.01 and ^*^p < 0.05 for 17-pt-PGE_2_ plus antagonist vs. antagonist alone; *ns*, not significant.

In addition to studying [Ca^2+^]_i_ in EP1^−/−^ neurons, separate experiments were conducted in WT neurons using SC-51089. Pre-treatment with SC-51089 (10 μM) reduced 17-pt-PGE_2_ (100 nM)-mediated [Ca^2+^]_i_ (AUC: 5.1 for 17-pt-PGE_2_ alone vs. 2.4 with SC-51089) (Figure 5A). Further, the effect of inhibiting the upstream COX-2 with NS-398 (10 μM) on [Ca^2+^]_i_ was studied using 17-pt-PGE_2_ (100 nM). In these experiments, NS-398 attenuated 17-pt-PGE_2_-mediated increase in [Ca^2+^]_i_ (AUC: 5.1 for 17-pt-PGE_2_ alone vs. 1.5 with NS-398) (Figure 5B). To determine the source of 17-pt-PGE_2_-induced [Ca^2+^]_i_, neuronal cultures were pre-treated with MK-801 (10 μM), L-type calcium channel blocker, nifedepine (10 μM) and ryanodine receptor (RyR) antagonist, ryanodine (50 μM). In all cases, application of the antagonists lead to a significant decrease of the 17-pt-PGE_2_-mediated [Ca^2+^]_i_ (Figures 5C–E). Application of all used antagonists alone induced small changes in [Ca^2+^]_i_ as compared to their respective baseline values. The 17-pt-PGE_2_-mediated change in [Ca^2+^]_i_ in the presence of NS-398 and ryanodine was not significantly different from the values in response to these antagonist alone. However, the 17-pt-PGE_2_-mediated change in [Ca^2+^]_i_ in the presence of MK-801 and nifedepine was significantly different from the values in response to these antagonist alone (*p* < 0.05, One-Way ANOVA with Bonferroni's multiple comparison tests). Following nifedepine treatment alone the measured [Ca^2+^]_i_ slightly but significantly decreased, the AUC has a negative value (−3.4) compared to that when the same antagonist was used with 17-pt-PGE_2_ (AUC: 0.38). This decrease in [Ca^2+^]_i_ by nifedepine treatment alone may have been due to the steady-state blockade of calcium influx via the L-type calcium channel.

### Hemin-induced changes [Ca^2+^]_i_ is EP1 and RyR-mediated

**Figure 6:**
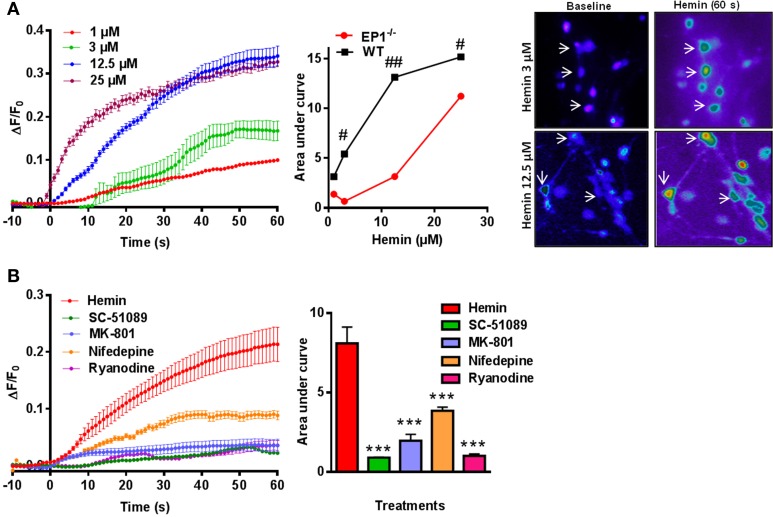
**Hemin mediated changes in [Ca^2+^]_i_ in neurons. (A)** Δ *F/F*_0_ induced by hemin (1, 3, 12.5, and 25 μM) in WT neurons. Plot represents relationship between extracellular concentration of hemin and [Ca^2+^]_i_ in WT and EP1^−/−^ neurons. Sequential pseudo color images of representative neurons shows changes in [Ca^2+^]_i_ in response to hemin (3 and 12.5 μM) measured using Fluo-8 AM (baseline and hemin for 60 s) in WT neurons. ^#^*p* < 0.01; ^##^*p* < 0.01 compared to the effect of hemin (3, 12.5, and 25 μM) in neurons cultured from WT and EP1^−/−^ mice. **(B)** Δ *F/F*_0_ induced by hemin (12.5 μM) alone and following pre-treatment with SC-51089 (10 μM), MK801 (10 μM), nifedepine (10 μM), and ryanodine (50 μM). Changes in AUC show that hemin-induced changes in [Ca^2+^]_i_ can be reduced by SC-51089, MK-801, nifedepine, and ryanodine. Data represent mean ± SEM. Statistical analysis was carried out using One-Way ANOVA, with Bonferroni's multiple comparison tests. ^***^p < 0.001 vs. hemin alone.

Previous reports suggest that iron neurotoxicity is mainly due to glutamate excitotoxicity (Munoz et al., 2011; Im et al., 2012). Hemin is an iron containing compound and iron is a direct product of hemin degradation and therefore we measured hemin-mediated changes in [Ca^2+^]_i_. Thus, following application of hemin (1–25 μM) for 60 s, we measured a dose-dependent increase in [Ca^2+^]_i_ and AUC in WT neuronal cultures (Figure 6A). Similar experiments in neuronal cultures from EP1^−/−^ mice resulted in a significant decrease in [Ca^2+^]_i_ compared to WT neuronal cultures. Pseudocolor microphotographs show changes in fluorescence in representative neurons before and after hemin treatment in WT neurons. The plot represents the relationship between extracellular concentrations of hemin and [Ca^2+^]_i_ in WT and EP1^−/−^ neurons. A hemin mediated increase in [Ca^2+^]_i_ in WT neurons was significantly different from that measured in EP1^−/−^ neurons (Figure 6A). Hemin treatment at 3, 12.5 and 25 μM in EP1^−/−^ neurons resulted in a significant decrease in the AUC than the AUC measured in WT neurons at the same concentrations of hemin. For example, at 12.5 μM hemin, the AUC in WT neurons was 13.13 and in EP1^−/−^ neurons it was 3.14 (*p* < 0.05). Using antagonists of the EP1 receptor, NMDAR and RyR, we found that hemin-induced [Ca^2+^]_i_ decreased. Treatment with SC-51089 (10 μM) (AUC: hemin 8.09 vs. SC-51089 0.90), MK-801 (10 μM) (AUC: 1.96), or ryanodine (50 μM) (AUC: 1.1) significantly decreased or abolished hemin-induced increase in [Ca^2+^]_i_. However, treatment with nifedepine (10 μM) (AUC: 3.8) only decreased hemin-induced [Ca^2+^]_i_ to 64.1 ± 16.6% of the average value following hemin only (Figure 6B). Altogether, these data suggest that in addition to the over activation of NMDAR's, hemin-induced neurotoxicity is mediated at least in part by the over-activation of the EP1 receptor.

## Discussion

Prostaglandin-mediated inflammation and the accumulation of red blood cell metabolites such as iron and hemin have been recognized in the pathogenesis of stroke, Alzheimer and Parkinson diseases. However, our knowledge of how prostaglandins and hemin accumulation results in neuronal death is unclear. The results presented here show that hemin is neurotoxic and also affects Ca^2+^ signaling in neurons. We showed that hemin dose-dependently increased the release of LDH from neurons and this effect was significantly smaller in neurons cultured from EP1^−/−^ mice. Also, we showed that 17-pt-PGE_2_-mediated Ca^2+^ signaling in EP1^−/−^ neurons was significantly less than compared to that in WT neurons, however; the increase in Ca^2+^ signaling was still evident in EP1^−/−^ neurons. Agonist 17-pt-PGE_2_ is a synthetic analog of PGE_2_ and is often used as a selective EP1 receptor agonist (murine *p*ki = 7.9). However, 17-pt-PGE_2_ also shows considerable EP3 receptor (G_*α q*_-protein - Ca^2+^ signaling) agonism at higher concentrations and therefore may explain why there was still Ca^2+^ signaling in EP1^−/−^ neurons following treatment with 17-pt-PGE_2_ (Okada et al., 2000). Future experiments would include using even a more selective EP1 receptor agonist when commercially available. Also, we showed that in mouse neuronal cultures, the DNA copy of the EP3 receptor is greater than that of the EP1 receptor. However, estimating copies of DNA for each receptor type alone does not allow us to speculate the impact this may have on the function of these receptors *in vitro*. Further, if available in the future, experiments with improved commercially available antibodies (greater selectivity) against the EP1 receptor would allow us to determine the functional implications of higher levels of the EP3 receptor compared to that of the EP1 receptor *in vitro*. Together with cell viability and Ca^2+^ imaging experiments, we showed that SC-51089 and MK-801 significantly reduced hemin-induced neurotoxicity suggesting that activation of NMDAR and EP1 receptors are involved in hemin-induced neurotoxicity. Calcium imaging studies presented here also indicate that hemin stimulates RyR-operated Ca^2+^ release as the blockade of RyR resulted in the decrease of hemin-induced [Ca^2+^]_i_ in WT neurons.

Previous reports have suggested that prostaglandins and their cognate receptors are involved in mechanisms of neurodegeneration in cerebral ischemia and excitotoxic brain injury (Doré, 2006; Serrano et al., 2011). When activated, EP1 receptor couples to G_*αq*_-proteins, resulting in increased phosphatidyl inositol hydrolysis and the elevation of [Ca^2+^]_i_. The increase in [Ca^2+^]_i_ has an important role in the control of neuronal function and plasticity (Catterall et al., 2013). However, over-activation of EP1 receptors may also generate supraphysiological changes in [Ca^2+^]_i_ that exacerbate neuronal cell death (Kawano et al., 2006). Due to the increased [Ca^2+^]_i_, pharmacologic blockade of EP1 receptor could be a promising therapeutic intervention against neuronal injury (Gendron et al., 2005). We and others have shown that when the EP1 receptor is blocked or ablated the neuronal damage and outcomes following ischemic stroke are improved (Ahmad et al., 2006a; Kawano et al., 2006; Saleem et al., 2007; Besancon et al., 2008; Abe et al., 2009; Zhen et al., 2011). Recently, it was also found that the death of cortical neurons induced by hypoxia was enhanced by activation of EP1 receptors *in vitro* (Liu et al., 2012). The role of [Ca^2+^]_i_ in neuronal activity and excitotoxicity following hemorrhage is unknown; however, data suggest that increased [Ca^2+^]_i_ contributes to increased cerebral vasospasm commonly seen in intracerebral hemorrhage (Kikkawa et al., 2010; Koide et al., 2011). However, protection of the brain against intracerebral hemorrhage following the blockade of the EP1 receptor may be complicated by the expression and signaling of the EP1 receptor in different cell types. For example, microglia cultured from EP1^−/−^ mice showed reduced neuroprotection when treated with NMDA in neuron-glia co-cultures (Carlson et al., 2009). Even though our *in vitro* data demonstrates that EP1 deletion provides neuroprotection against hemin-induced cell death, recently our group has found that EP1 deletion may exacerbate neurobehavioral impairments in a collagenase-induced mouse model of hemorrhagic stroke by impairing the function of microglial cells surrounding the area of injury (Singh et al., 2013). The differences in the function of the EP1 receptors between *in vitro* and *in vivo* models are so numerous that different cell types that express the EP1 receptor may reflect the overall outcomes. Although the hemin-mediated increase in [Ca^2+^]_i_ was significantly different between WT and EP1^−/−^ neuronal cultures, the EP1 receptor pathway may not be the single excitotoxic pathways activated by hemin.

The overactivation of postsynaptic glutamate receptors and the neuronal loading of Na^+^ and Ca^2+^ results in the rapid depletion of neuronal energy, with a significant reduction in adenosine triphosphate (Tsuji et al., 1994; Atlante et al., 1996). Similarly, neuronal cell death in preclinical models of stroke has also been linked to NMDAR overactivation (Besancon et al., 2008). The increase in transient levels of extracellular glutamate has been postulated to trigger rapidly evolving free radical production through the activation of Ca^2+^-permeable NMDARs (Lafon-Cazal et al., 1993; Reynolds and Hastings, 1995; Thiex et al., 2007). Also, the activation of synaptic or extrasynaptic NMDARs has been reported to be neuroprotective or excitotoxic, respectively (Hardingham et al., 2002; Stark and Bazan, 2011). Interestingly, Bazan's group also suggested that cell death following stimulation of extrasynaptic NMDARs is due to the induction of COX-2 signaling from a physiological to a potentially pathological process. In an separate earlier study, activation of the EP1 receptor was found to disrupt Ca^2+^ homeostasis with NMDA treatment by impairing the function of Na^+^/Ca^2+^ exchanger mechanisms (Kawano et al., 2006). To demonstrate the role of NMDARs in hemin neurotoxicity, we used the NMDAR antagonist MK-801, and found that it significantly reduced hemin neurotoxicity and hemin-mediated increase in [Ca^2+^]_i_. The measured decrease in hemin neurotoxicity and [Ca^2+^]_i_ after blockade of NMDARs suggests that NMDAR activation is also involved and perhaps more so during the initial phases of hemin-induced [Ca^2+^]_i_ in neurons. Interestingly, our data also show that the hemin-induced increase in [Ca^2+^]_i_ was partially decreased with nifedepine, a selective blocker of L-type Ca^2+^ channels. The involvement of Ca^2+^ channels in hemin neurotoxicity is possibly due to presynaptic facilitation of glutamate release and consequent overactivation of NMDARs. Considering the predominate effects of hemin on NMDAR-mediated neuronal death, we postulate that Ca^2+^ entry via postsynaptic and/or extrasynaptic NMDARs may alter the neuronal excitability and cellular signaling resulting in a deficit in overall neuronal networks.

Iron, as a neurotoxic breakdown product of heme and Hb, has previously been shown to stimulate RyR and mediate an increase in [Ca^2+^]_i_ through its capacity to generate ROS (Munoz et al., 2006; Hidalgo et al., 2007). Also in support, recent data showed that blockade of L-type calcium channels protected hippocampal neurons against iron neurotoxicity *in vivo* (Bostanci and Bagirici, 2013). Taken together (these facts and our experimental data), we speculate that hemin may also stimulate RyR-mediated Ca^2+^ release through its capacity to generate ROS. In this study, following treatment with RyR antagonist, hemin-induced increase in [Ca^2+^]_i_ was abolished suggesting that RyR is involved in the hemin-induced increase in [Ca^2+^]_i_ following stimulation of NMDARs. These data show that hemin-induced [Ca^2+^]_i_ signaling is rapidly initiated and is a lasting process that may have a role in neuronal death.

In summary, we have demonstrated that pharmacologic blockade and genetic deletion of the EP1 receptor can affect the outcome of hemin-induced cell death in cultured postnatal cortical neurons. Our data suggest that hemin-induced neurotoxicity is mediated by an abnormal increase in [Ca^2+^]_i_ via activation of multiple mutually dependent processes involving NMDARs, L-type Ca^2+^ channels, and calcium-induced calcium release via activation of intracellular RyR and COX-2 pathway. Also, the data that 17-pt-PGE_2_ and hemin-mediated increase in [Ca^2+^]_i_ were inhibited by blockade of the EP1 receptor suggest involvement of this receptor as an important counterpart in hemin-induced toxicity.

### Conflict of interest statement

The authors declare that the research was conducted in the absence of any commercial or financial relationships that could be construed as a potential conflict of interest.
